# An Atlantic-driven rapid circulation change in the North Pacific Ocean during the late 1990s

**DOI:** 10.1038/s41598-019-51076-1

**Published:** 2019-10-08

**Authors:** Chau-Ron Wu, Yong-Fu Lin, You-Lin Wang, Noel Keenlyside, Jin-Yi Yu

**Affiliations:** 1 Department of Earth Sciences, National Taiwan Normal University, Taipei, Taiwan; 2 Geophysical Institute, University of Bergen and Bjerknes Centre for Climate Research, Bergen, Norway; 3 Department of Earth System Science, University of California at Irvine, Irvine, California USA

**Keywords:** Climate-change impacts, Physical oceanography

## Abstract

Interbasin interactions have been increasingly emphasized in recent years due to their roles in shaping climate trends and the global warming hiatus in the northern hemisphere. The profound influence from the North Atlantic on the Tropical Pacific has been a primary focus. In this study, we conducted observational analyses and numerical modeling experiments to show that the North Atlantic has also strongly influenced the Extratropical North Pacific. A rapid and synchronous change in the atmospheric and oceanic circulations was observed in the North Pacific during the late 1990s. The change was driven by the transbasin influence from the Atlantic Ocean. During the positive phase of the Atlantic Multidecadal Oscillation (AMO) since the 1990s, the anomalously warm North Atlantic triggers a series of zonally symmetric and asymmetric transbasin teleconnections involving the Inter-tropical Convergence Zone (ITCZ), Walker and Hadley circulations, and Rossby wave propagation that lead to a decrease in wind stress curls over the Pacific subtropics, resulting in an abrupt weakening in the North Pacific subtropical gyre (NPSG) and the Kuroshio Current.

## Introduction

Many lines of evidence demonstrate that the climate system has been warming since the last century. This warming was interrupted during a period from the late 1990s to 2012–2013 that is referred to as the global warming hiatus^1,2^. Drastically different impacts of the warming and the hiatus have been observed on the global atmospheric and oceanic circulations. In the Pacific, for example, the Kuroshio appears to have intensified over the last century^3^ but weakened during the hiatus period^4^. The atmospheric circulation patterns associated with the hiatus should have also caused changes in other aspects of the Pacific Ocean circulation^5^.

**Figure 1:**
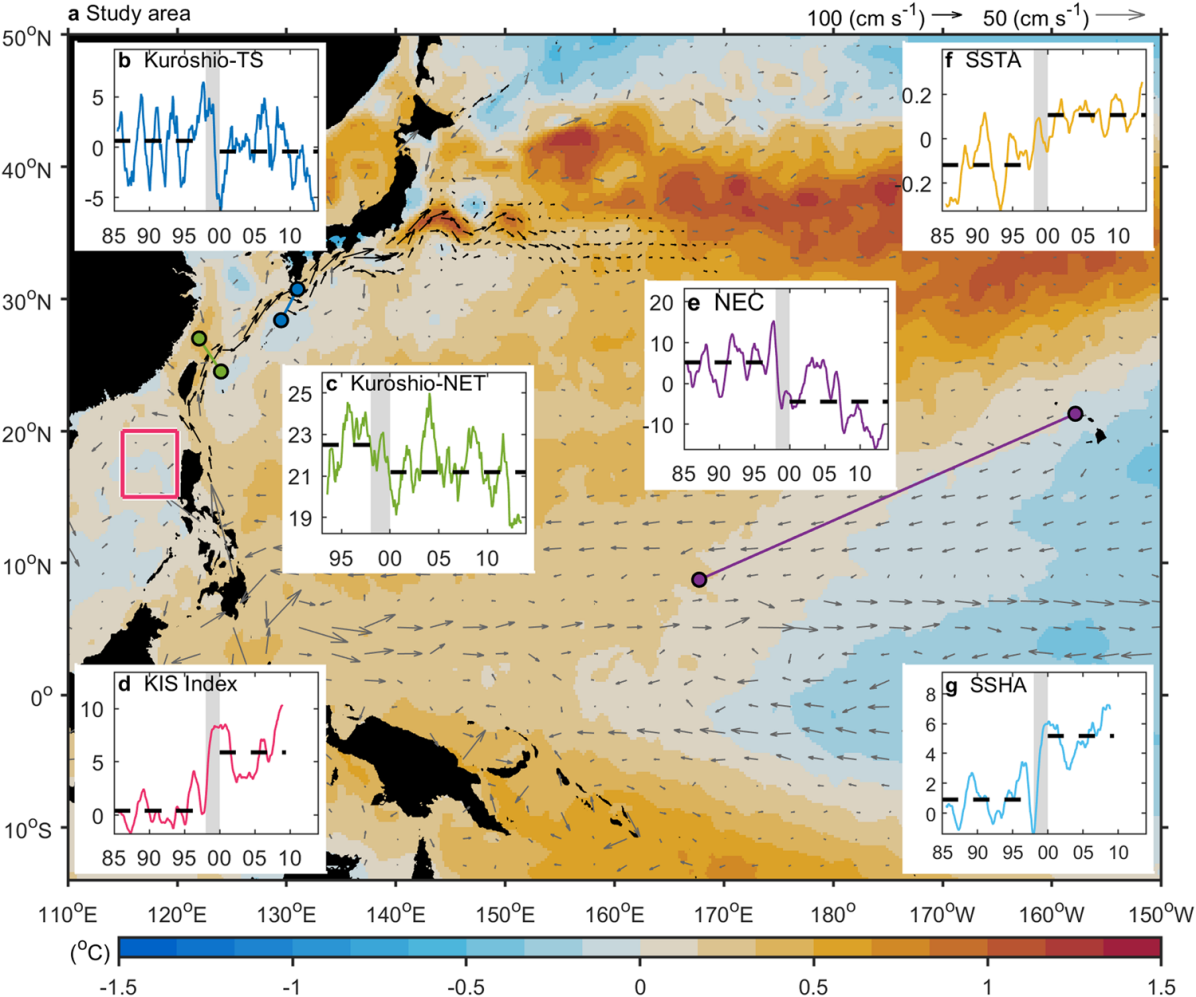
Ocean circulation characteristics in the western North Pacific. (**a**) SST difference (shading) (1999–2013 minus 1993–1998) and mean geostrophic velocities (vectors) in the study area. (**b**) Monthly sea level anomaly difference (in units of cm) between the Naze and Nishinoomote tidal gauges (blue dots in **a**) across Tokara Strait, denoting the Kuroshio intensity. (**c**) The Kuroshio upper layer transport in the northeast Taiwan (NET, green dots in Fig. 1a) (in unit of Sv). (**d**) Index of the Kuroshio intrusion into the SCS obtained by averaging SSHA in the region of 115–120°E and 15–20°N. (**e)** Monthly SSHA (in units of cm) between the Honolulu and Kwajalein tidal gauges (orange dots in **a**) across the NEC. (**f)** Monthly SSTA (in units of °C) over the western North Pacific (110°E–150°W and 14°S–50°N). (**g**) Same as (**f**), but for SSHA (in units of cm). In (**b–g**), the gray shading indicates the period of 1998–99, black dashed lines indicate mean values.

Figure 1 shows the ocean circulation and temperature patterns in the northwestern Pacific during the global warming hiatus. The Kuroshio flows northward along the coasts of Luzon and Taiwan, entering the East China Sea through the passage off northeast Taiwan, and continues roughly along the continental slope to the northeast. The Kuroshio south of Japan enters the Pacific through the Tokara Strait and finally becomes the North Pacific Current east of Japan. Based on geostrophy, the sea level difference (SLD) across the Kuroshio is a useful proxy for its intensity^6,7^. Several estimates of Kuroshio intensity have been made using the SLD from tide gauge measurements in the Tokara Strait^6^. Using a similar approach, we derived the decadal changes in the surface intensity of the Kuroshio since 1985. The monthly SLD between the Naze and Nishinoomote tidal gauges (which straddle the Tokara Strait) exhibits year-to-year variations, with evidence of a drop around 1998–99 (Fig. 1b). The altimeter-derived Kuroshio transport northeast of Taiwan shows a similar decrease around 1998–99 (Fig. 1c).

A much more abrupt change in 1998–99 is apparent in the time series of a sea level index representing a Kuroshio intrusion into the South China Sea (SCS)^8^ (Fig. 1d). The generally negative (low) sea levels west of Luzon Island indicate that the Kuroshio tended to intrude into the SCS prior to 1998. In contrast, the post-1998 era is characterized by positive sea levels west of Luzon Island, indicating that the Kuroshio has tended to bypass the Luzon Strait without significant westward encroachment. Figure 1e shows the transport of the North Equatorial Current (NEC) derived from the sea level difference between Honolulu and Kwajalein. Wyrtki^9^ demonstrated that the NEC transport calculated using these two tidal stations is accurate. A weakening tendency in the surface NEC is observed during the post-1998 era. Analyses of sea-surface temperature anomalies (SSTAs) and sea-surface height anomalies (SSHAs) averaged over the western North Pacific further support a drastic alteration in ocean properties around 1998–99 (Fig. 1f,g).

**Figure 2:**
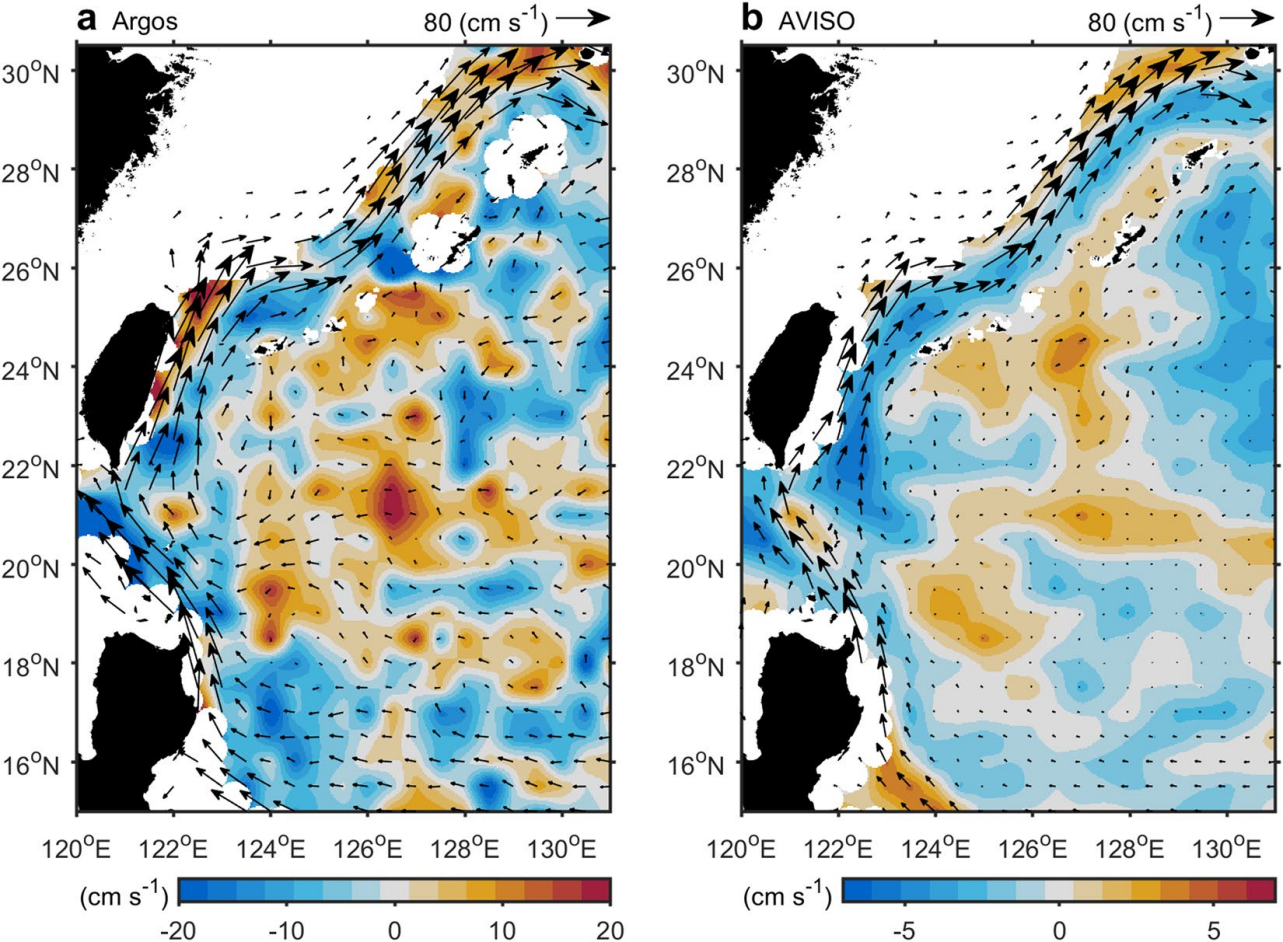
Surface velocity differences between the two periods. Monthly velocity anomaly differences (shading) (1999–2013 minus 1993–1998) from (**a**), Argos and (**b**), AVISO data sets. Vectors indicate long-term mean velocity.

To further investigate the changes in the ocean circulation in the western North Pacific, we divided the time interval 1993–2013 into two periods: 1993–1998 and 1999–2013. Five different datasets were used to calculate surface velocity differences between the two periods, Argo drifters, altimetry observations from the Archiving, Validation and Interpretation of Satellite Oceanographic (AVISO) satellite, and three ocean reanalysis products (HYCOM, JCOPE-2, and GODAS) that are independent of each other in their assimilation methods and simulation settings. Figure 2a,b show the surface velocity differences in the Kuroshio region based on Argo drifters and altimeter-derived geostrophic currents, respectively. Both exhibit negative values (shown in blue band) along the main stream of the Kuroshio. A weakened Kuroshio is also evident in all the three reanalysis products (Fig. S1).

**Figure 3:**
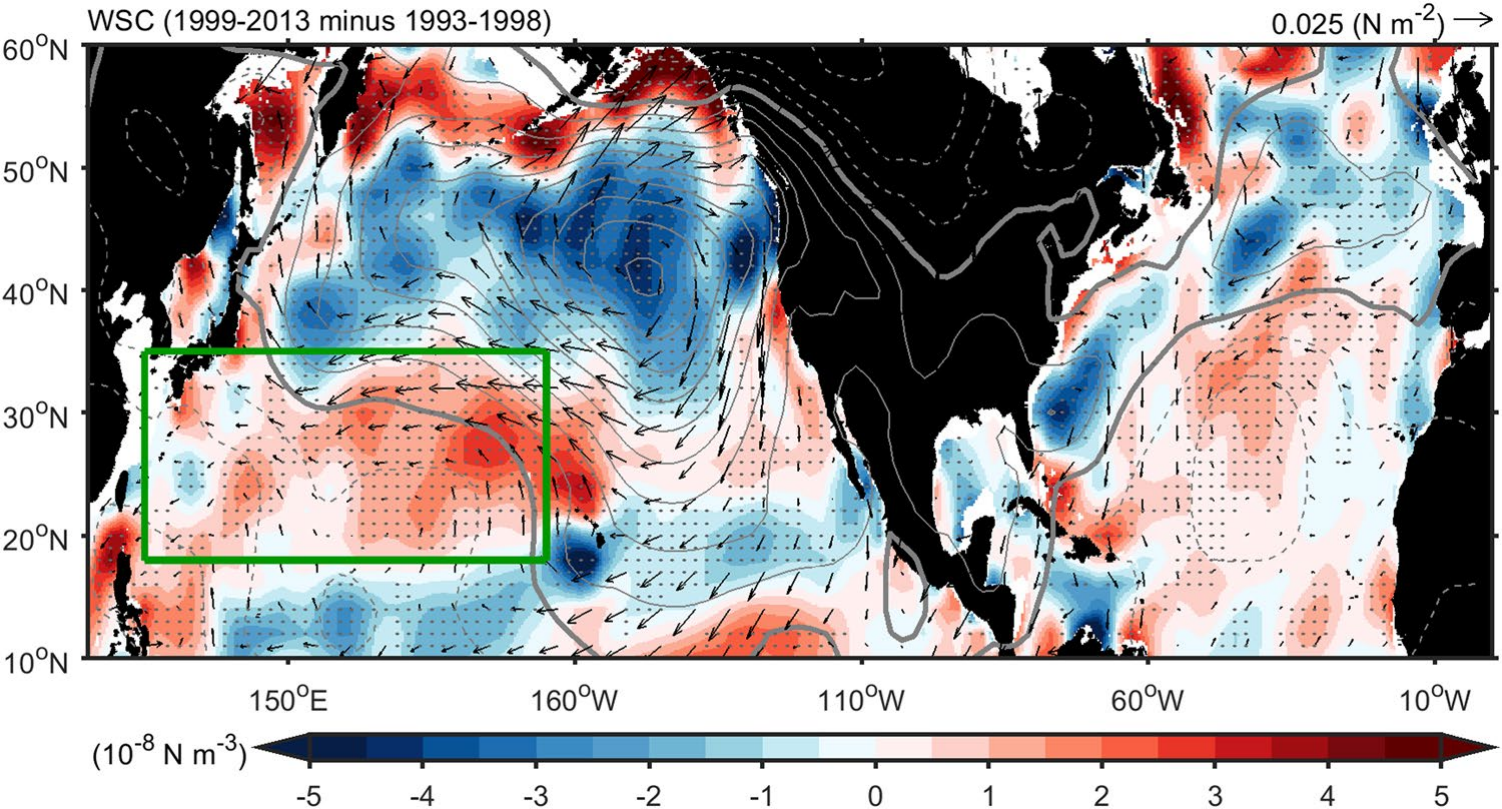
Monthly wind stress and wind stress curl anomalies. Ensemble average of monthly sea level pressure (contour), wind stress (vector) and wind stress curl anomaly (WSCA) difference (shading) (1999–2013 minus 1993–1998) from various data sets (NCEPr1, NCEPr2, ERAint, and JRA55). Gray dots indicate where results of WSCA difference are consistent in those data set. The green box indicates the region of the subtropical gyre (125°E–165°W; 18–35°N).

Surface ocean circulation variability is usually associated with variability in the surface wind field^4^. Figure 3 shows the changes of surface wind stress (vector) and wind stress curl anomaly (WSCA; color) between the two periods averaged from four atmospheric reanalysis products. A weakening of surface westerlies is evident over the North Pacific during the 1999–2013 period in this multi-analysis mean as well as in each individual reanalysis (Fig. S2). The weakened westerlies result in positive WSCAs in the subtropics, which should weaken the North Pacific subtropical gyre (NPSG), and subsequently the Kuroshio^4^. There are also positive WSCAs over the North Pacific that should act to weaken the subpolar gyre and in turn the western boundary currents. The mechanism responsible for the wind changes warrants further examination.

To further confirm and examine oceanic and atmospheric changes in the North Pacific around 1998–99, we have extended the first period from 1984 to 1998 based on various data sets for statistical tests. Table S1 and Fig. S3 summarize differences for ocean circulation characteristics, while Fig. S4 shows differences for atmospheric parameters. All differences are statistical significance above the 99% confidence level based on t-test, indicating drastic changes in oceanic and atmospheric environments take place around 1998–99.

Recent studies^10–13^ have discovered that the Atlantic Ocean has acted as a pacemaker for global climate in recent decades, contributing to the global warming hiatus and multidecadal fluctuations in the global mean surface temperature, especially in the Northern Hemisphere. The Atlantic Multidecadal Oscillation (AMO), with a period of 65–80 years, is the leading mode of decadal variability mode in the Atlantic Ocean that exerts profound impacts not only on the Atlantic and North American climate but also the Pacific climate variability^14,15^, including the Southeast and East Asian summer monsoons^16^ and El Niño and South Oscillation (ENSO)^17^.

**Figure 4:**
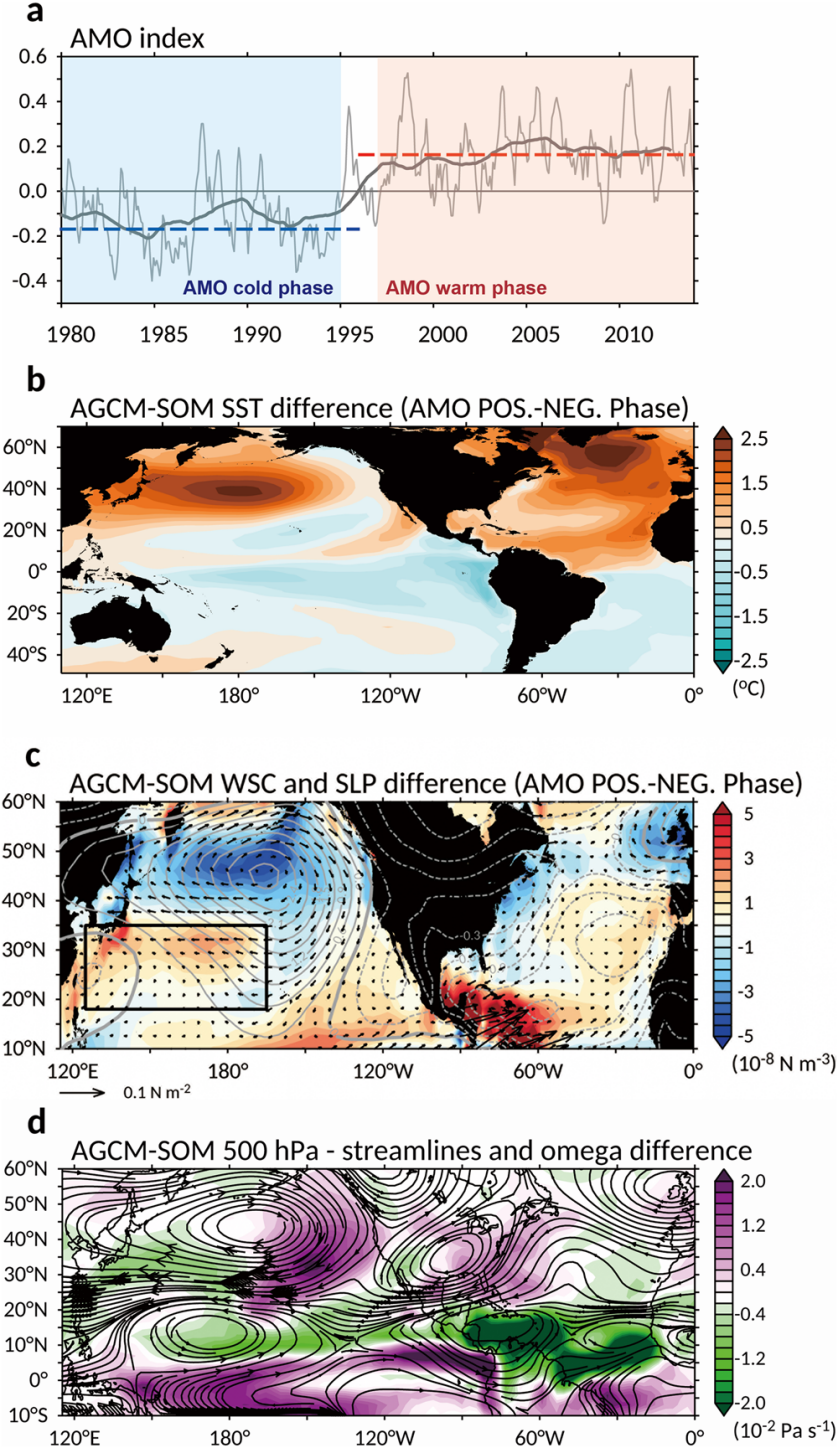
The AMO-related sea surface temperature and wind field. (**a)** Monthly time series of AMO index (in units of °C, thin line). The bold line shows the AMO index with 61-month running mean. The AMO positive (negative) periods are marked by the red (blue) shading. Red (blue) dashed lines indicate mean of the AMO positive (negative) periods. The AMO-related (**b)**, sea surface Temperature (SST), (**c)**, sea level pressure (contour), wind stress (vector), wind stress curl (WSC), and (**d)**, 500 hPa streamlines and vertical velocities from the AGCM-SOM experiment, calculated as differences between the AMO positive and negative runs of the model. The solid/dash contours denote positive/negative anomaly. Note that in (**b**–**d**), the North Atlantic SSTs were prescribed based on the regressions onto the AMO index. The black box indicates the region of the subtropical gyre (125°E–165°W; 18–35°N).

Figure 4a displays the time series of the AMO index during the period 1980–2013, and shows that the AMO shifted to its positive phase in the mid-1990s. This phase change time is a few years before the abrupt changes in Pacific Ocean circulation were observed (as shown in Fig. 1). Previous studies observed baroclinic responses in the western subtropical gyre and Kuroshio Extension relative to atmospheric forcing using historical hydrography and satellite altimetry data^18–21^. As mentioned, it takes a few years for surface wind pattern changes to induce upper ocean circulation changes. Therefore, it is possible that the mid-1990s phase change in the AMO can be a cause for the late-1990s changes in the Pacific Ocean circulation. To examine this possible transbasin influence of the Atlantic on Pacific, we performed numerical experiments with the NCAR Community Atmospheric Model, version 3.0, (CAM3.0)^22^ coupled to a mixed-layer slab ocean model (SOM). SSTAs were prescribed in the North Atlantic (0–70° N) to represent the positive AMO phase in one experiment (i.e., AMO-positive experiment) and the negative AMO phase in the other experiment (i.e., AMO-negative experiment). The slab ocean is free to interact with the CAM3 model in other ocean basins.

**Figure 5:**
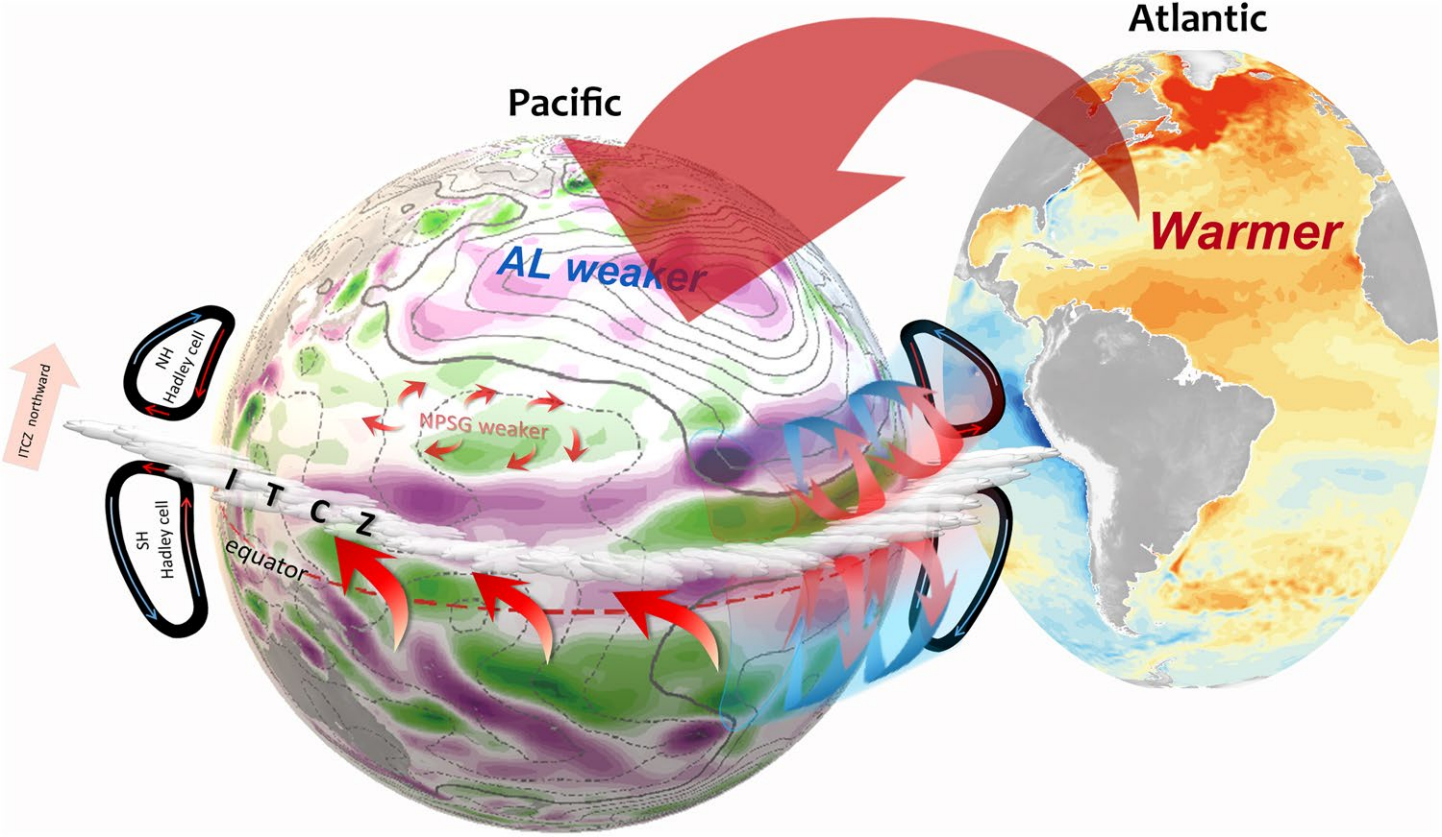
Diagram of the linkage between the Atlantic and North Pacific. The anomalously warm North Atlantic and cold South Atlantic (right) leads to weakened Hadley cell in the Northern Hemisphere but strengthened Hadley cell in the Southern Hemisphere, resulting in a northward displacement of the ITCZ not only in the Atlantic but also in the Pacific. The weakened Hadley cell leads to a positive WSCA in the Pacific subtropical region, resulting in a weakened NPSG (left). The tropical North Atlantic warming associated with the positive AMO phase can also trigger a zonally-asymmetric circulation mechanism to weaken the NPSG. The Atlantic warming can induce an anomalous Walker circulation that descends over the tropical central Pacific and suppresses deep convection there, the anomalous cooling resulting from which can then excite a Rossby wave response to higher latitudes inducing a low-level anomalous cyclone and low pressure over the subtropical Pacific, and an anomalous high pressure over the North Pacific (i.e. Aleutian Low weaker, contours), which also result in a weakened NPSG and Kuroshio. The colors are wind stress curl (left, in units of 10^−8^ N m^−3^) and SST (right, in units of °C) difference (1999–2013 minus 1993–1998) from NCEP r2 and AVHRR OISST data. The green/purple colors denote the cyclonic/anticyclonic anomaly (left). The contours are sea level pressure (left, in units hPa) difference (1999–2013 minus 1993–1998) from NCEP r2. The solid/dash contours denote positive/negative anomaly. The arrows indicate the surface wind field and atmospheric meridional overturning circulation (left). The AL denotes the Aleutian Low.

Figure 4b shows the SST differences between the two experiments. In the North Atlantic, the SSTA differences resembles the observed AMO pattern^23^, which is characterized by a tropical and a subpolar warming bands separated by a weaker-warming band in between (Fig. 5). Figure 4c shows wind stress and wind stress curl differences between the two experiments. The weakened westerlies and positive WSCA in the subtropical region resemble those observed during 1999–2013 (cf. Fig. 3). The pattern correlations over the subtropical Pacific between the model differences and the observed changes in Fig. 3 are 0.49 (P < 0.01) for zonal surface wind anomalies and 0.27 (P < 0.01) for WSCAs. This modeling result adds further support to the suggestion that the AMO phase change is likely a cause for the decelerating westerlies and positive WSCA in the subtropical Pacific region that results in a weakened NPSG and Kuroshio during 1999–2013.

The transbasin influence of the AMO on the Pacific can be explained by zonally-symmetric as well as zonally-asymmetric circulation mechanisms in the atmosphere^24^. The zonal-symmetric circulation mechanism is based on the fact that the Inter-tropical Convergence Zone (ITCZ)^25,26^ typically displaces toward the warmer hemisphere^27,28^ where the Hadley cell is weaker due to the smaller meridional gradient in surface temperatures^29^. The weaker Hadley cell results in a weaker subtropical high and weaker surface westerlies to the north of the high due to geostrophic balance or reduced eddy transport in the mid-latitudes^27,30^. During the positive AMO phase, an anomalously warm North Atlantic and cold South Atlantic should displace the ITCZ northward and weakened the Northern Hemisphere Hadley not only in the Atlantic but also in the Pacific. A Pacific ITCZ index based on the precipitation anomalies in the northwestern tropical Pacific (averaged over 0–10°N and 130–160°E)^31^ confirms that the Pacific ITCZ is displaced southward during the negative AMO phase of the pre-1990 era but northward during the positive AMO phase of the post-1990 era (Fig. S5)^24^. The zonal-mean Hadley circulation also became weaker in the post-1990 period and in the positive AMO phase experiment not only over the North Atlantic but also over the North Pacific (Fig. S6).

The tropical North Atlantic warming associated with the positive AMO phase can also trigger a zonally-asymmetric circulation mechanism that acts to weaken the Pacific subtropical high^32^. In this mechanism, the Atlantic warming induces an anomalous Walker circulation with a descending branch over the tropical central Pacific thus suppressing convection^33–37^. The resultant can then excite a Rossby wave response to higher latitudes inducing an anomalous cyclone over the subtropical Pacific, an anomalous anti-cyclone over the North Pacific, and an anomalous cyclone over the North America, which are not only in upper-level also in low-level. This wavetrain pattern of anomalies in the low-level and upper-level circulation is clearly evident in the positive AMO experiment (Fig. 4c,d) and can also explain the weakened subtropical Pacific high, Aleutian Low, and mid-latitude surface westerlies observed during the post-1990 period (Fig. 3).

As schematized in Fig. 5, our observational analyses and climate model experiments suggest that the change of the AMO to a positive phase in the middle 1990s can trigger a series of zonally symmetric and asymmetric transbasin processes to give rise to a late-1990s abrupt change of the North Pacific circulation that is manifested as a weakening of the NPSG and Kuroshio. This Atlantic or AMO control of the North Pacific circulation was not noted in earlier periods of instrumental observations. This can be an indication of an emerging change of climate dynamics due to global warming that deserves attention.

## Materials and Methods

### Observations and reanalysis data

The monthly-mean SSTs of the ERSST (Extended Reconstructed Sea Surface Temperature, version 5) were provided by the NCEI/NOAA (National Centers for Environmental Information/National Oceanic and Atmospheric Administration, https://data.nodc.noaa.gov) with 2° × 2° horizontal resolution since 1854^38^. The daily Advanced Very High Resolution Radiometer – Optimum Interpolation SST (AVHRR-OISST, http://www.ncdc.noaa.gov/oisst) data on a global 0.25° grid were used for Fig. 5. The 6-hourly ocean surface velocity at 15 m was provided by the GDP (Global Drifter Program, http://www.aoml.noaa.gov) /NOAA beginning in 1979^39^. The tide gauge data were provided by the UHSLC (University of Hawaii Sea Level Center, https://uhslc.soest.hawaii.edu/)^40^. The SSHAs of the Reconstructed Sea Level dataset (version 1) were provided from CCAR (Colorado Center for Astrodynamics Research, the University of Colorado) and distributed by the JPL/NASA (Jet Propulsion Laboratory/National Aeronautics and Space Administration, https://podaac.jpl.nasa.gov/) since 1958, with 0.5 degree in spatial resolution and 7-day in temporal resolution^41^. The daily absolute geostrophic velocity (GSV) products (version: DT-MADT two-sat) were produced by Ssalto/Duacs and distributed by the AVISO (Archiving, Validation and Interpretation of Satellite Oceanographic Data, http://www.aviso.altimetry.fr) on a global 0.25° grid since 1993. The volume transport of the upper-ocean Kuroshio (shown in Fig. 1c) is calculated as $\int v\cdot D\,dm\,$, where *ν* is the velocity across the Kuroshio axis, *dm* is the distance between two neighboring stations (122°E, 27°N; 124°E, 24.5°N) across the Kuroshio (shown in Fig. 1a), and *D* is the mean depth (400 m) of the upper-ocean (modified from Hwang and Kao^42^).

Four atmospheric reanalysis products were used, the NCEPr1 (National Centers for Environmental Prediction/National Center for Atmospheric Research reanalysis 1)^43^, the NCEPr2 (National Centers for Environmental Prediction/Department of Energy AMIP reanalysis2)^44^ (http://www.cpc.ncep.noaa.gov/), the ERAint (European Center for Medium-Range Weather Forecasts Reanalysis Interim, https://www.ecmwf.int/)^45^, and the JRA55 (Japanese 55-year Reanalysis, http://jra.kishou.go.jp/)^46^. The monthly NCEPr1 and NCEPr2 products are provided on a global 1.875° grid for the years 1948 (1979) to the present for the NCEPr1 (NCEPr2). The monthly ERAint product with an 80 km horizontal resolution is available from 1979 to the present. The monthly JRA55 was provided by the JMA (Japan Meteorological Agency), has a 55 km horizontal resolution and covers the period from 1958 to the present.

### Model simulations

Numerical model experiments were performed using the NCAR Community Atmospheric Model, version 3.0, (CAM3.0)^22^ with a T42 Eulerian spectral resolution (128 × 64 grid points) and 26 vertical levels. Model experiments were carried out with prescribed AMO-associated SSTs in the North Atlantic (NA) (0°–70°N). In this set of the experiments, the atmospheric general circulation model (AGCM) is coupled to a mixed layer slab ocean model (SOM) in other ocean basins and referred to as the AGCM-SOM experiments. The AGCM-SOM was integrated for 120 years for each of the two experiments, and model output from the last 100 years were used for the analysis. Using only the last 40 years of the AGCM- SOM output gives very similar results. The simulated AMO responses were defined as the mean state differences between the AMO-positive and AMO-negative experiments.

### Climate indices

The AMO index^47^ is obtained from the Physical Sciences Division (http://www.esrl.noaa.gov/psd/data/timeseries/AMO/) which is calculated as the detrended SSTAs averaged over the North Atlantic from the equator to the 70°N.

### Statistical analyses

The correlation of significance test is performed based on the two-tailed Student’s t-test. More analyses and discussion on the statistical significance have been included in Supplementary Information.

## Supplementary information

Supplementary Info
