# Supplementary material for: An Atlantic-driven rapid circulation change in the North Pacific Ocean during the late 1990s
Source: Sci Rep. 2019 Oct 8;9:14411. doi: 10.1038/s41598-019-51076-1 (PMC6783482; doi:10.1038/s41598-019-51076-1)
Supplement: Supplementary file 1 — Supplementary Info [file 41598_2019_51076_MOESM1_ESM.docx]

**An Atlantic-driven rapid circulation change in the North Pacific Ocean during the late 1990s**

Chau-Ron Wu^1,*^, Yong-Fu Lin^1,3^, You-Lin Wang^1^, Noel Keenlyside^2^, and Jin-Yi Yu^3^

^1^Department of Earth Sciences, National Taiwan Normal University, Taipei, Taiwan

^2^ Geophysical Institute, University of Bergen and Bjerknes Centre for Climate Research, Bergen, Norway

^3^Department of Earth System Science, University of California, Irvine, Irvine, California

*Correspondence to cwu@ntnu.edu.tw

**1. AMO impacts on the oceanic and atmospheric environments in the North Pacific**

Three different ocean reanalysis products (HYCOM, JCOPE-2, and GODAS) were used to calculate the surface velocity differences between the 1999-2013 and 1993-1998 periods. A weakened Kuroshio main stream is evident to various degrees in all three reanalysis products (Fig. S1).

Surface ocean circulation variability is usually associated with variability in the surface wind field. Figure S2 shows the differences in wind stress and wind stress curl anomaly (WSCA) between the 1999-2013 and 1993-1998 periods calculated from each of the four atmospheric reanalysis products. All four products are consistent in revealing weakened surface westerlies over the North Pacific during the later period (1999-2013). The weakened westerlies result in positive negative WSCAs over the subtropical Pacific, leading to a weakened North Pacific subtropical gyre (NPSG) and Kuroshio compared to the 1993-1998 period.

To further confirm and examine oceanic and atmospheric changes in the North Pacific around 1998-99, we have extended the first period from 1984 to 1998 based on various data sets for statistical tests. Table S1 and Fig. S3 summarize differences for ocean circulation characteristics, while Fig. S4 shows differences for atmospheric parameters. All differences are statistical significance above the 99% confidence level based on t-test, indicating drastic changes in oceanic and atmospheric environments take place around 1998-99.

**2. A precipitation-based ITCZ index**

An ITCZ index is used to quantify ITCZ location in the northwestern tropical Pacific^1^. The index is defined as the precipitation anomalies averaged between 0–10°N and 130–160°E. Figure S5 displays the variations in the ITCZ index during the analysis period.


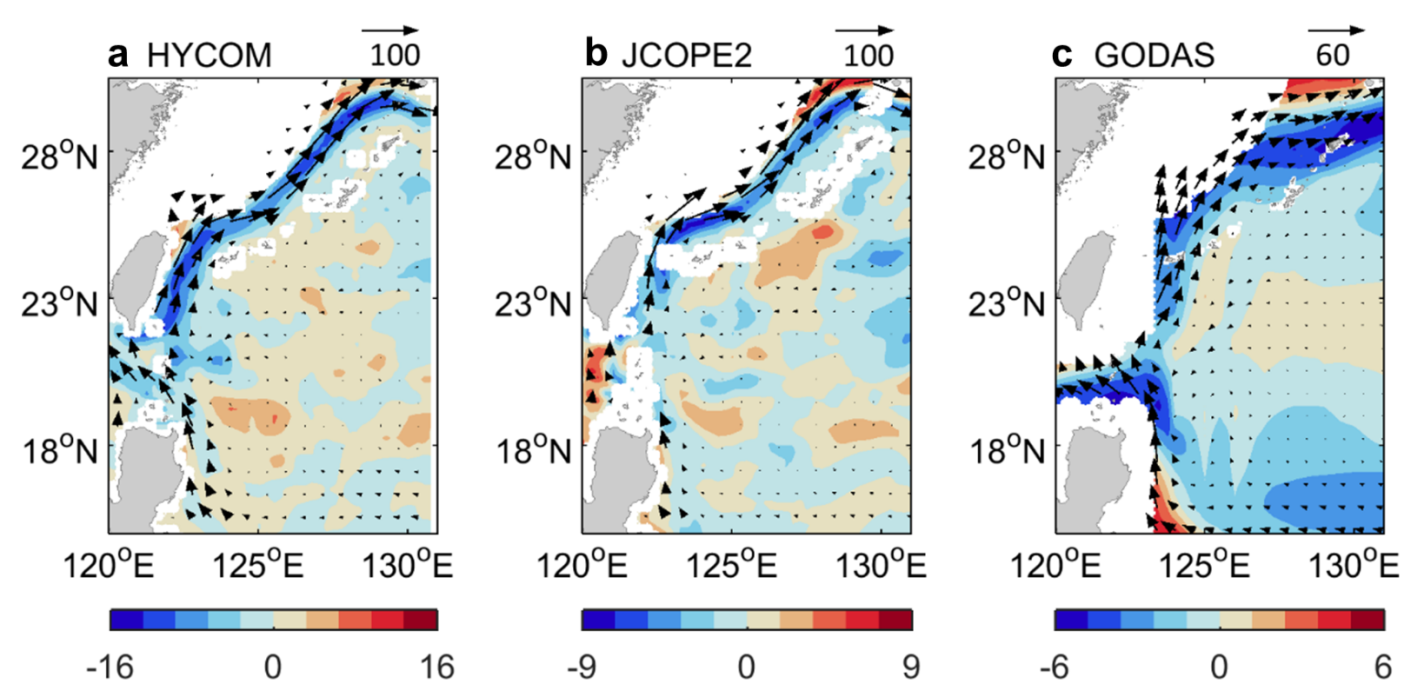


**Figure S1. Surface velocity differences before and after the 1998-99 abrupt change from three ocean reanalysis products.**

Monthly velocity anomaly differences (shading, in units of cm s^-1^) are calculated as the 1999-2013 values minus the 1993-1998 values. Vectors indicate the mean velocity (in units of cm s^-1^) averaged from 1993 to 2013.


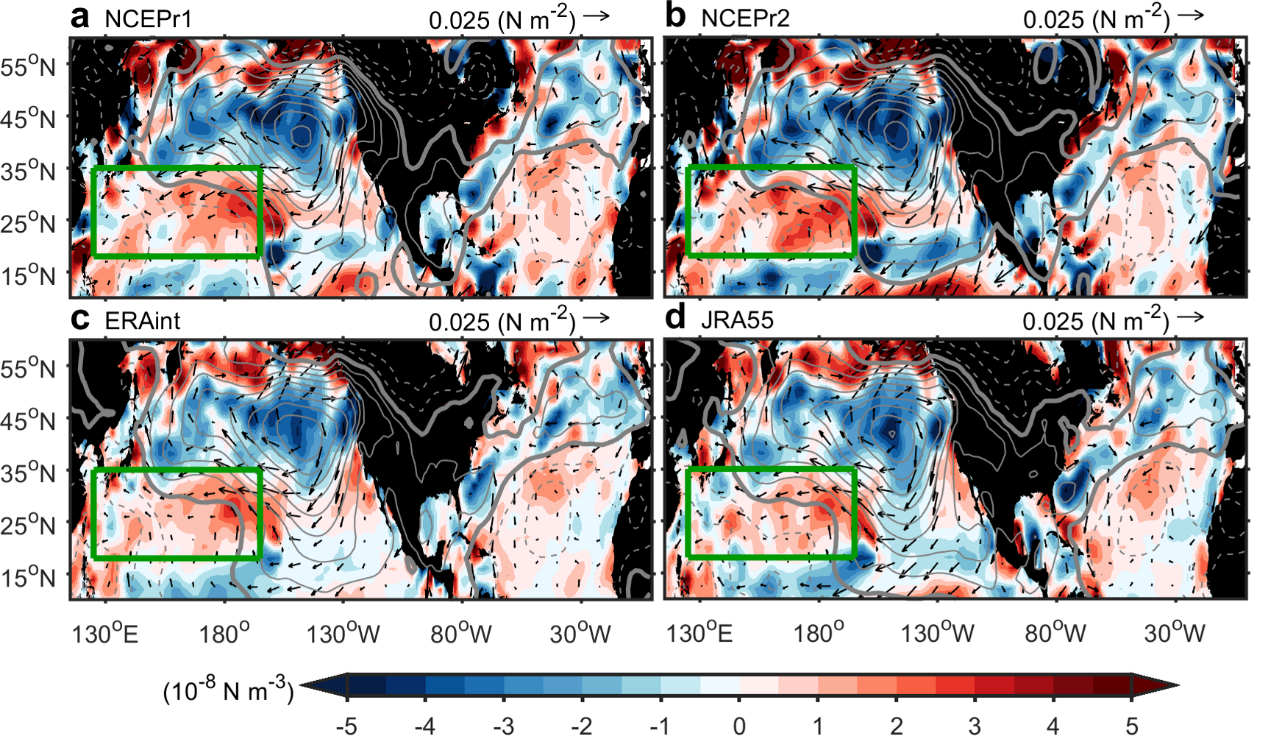


**Figure S2. Monthly sea level pressure (contour), wind stress (vector) and wind stress curl anomaly (shading, in units of 10^-8^ N m^-3^) changes from 1993-98 to 1999-2013 calculated from four atmospheric reanalysis products.**

The green box indicates the location of the subtropical gyre (125°E-165°W; 18-35°N).


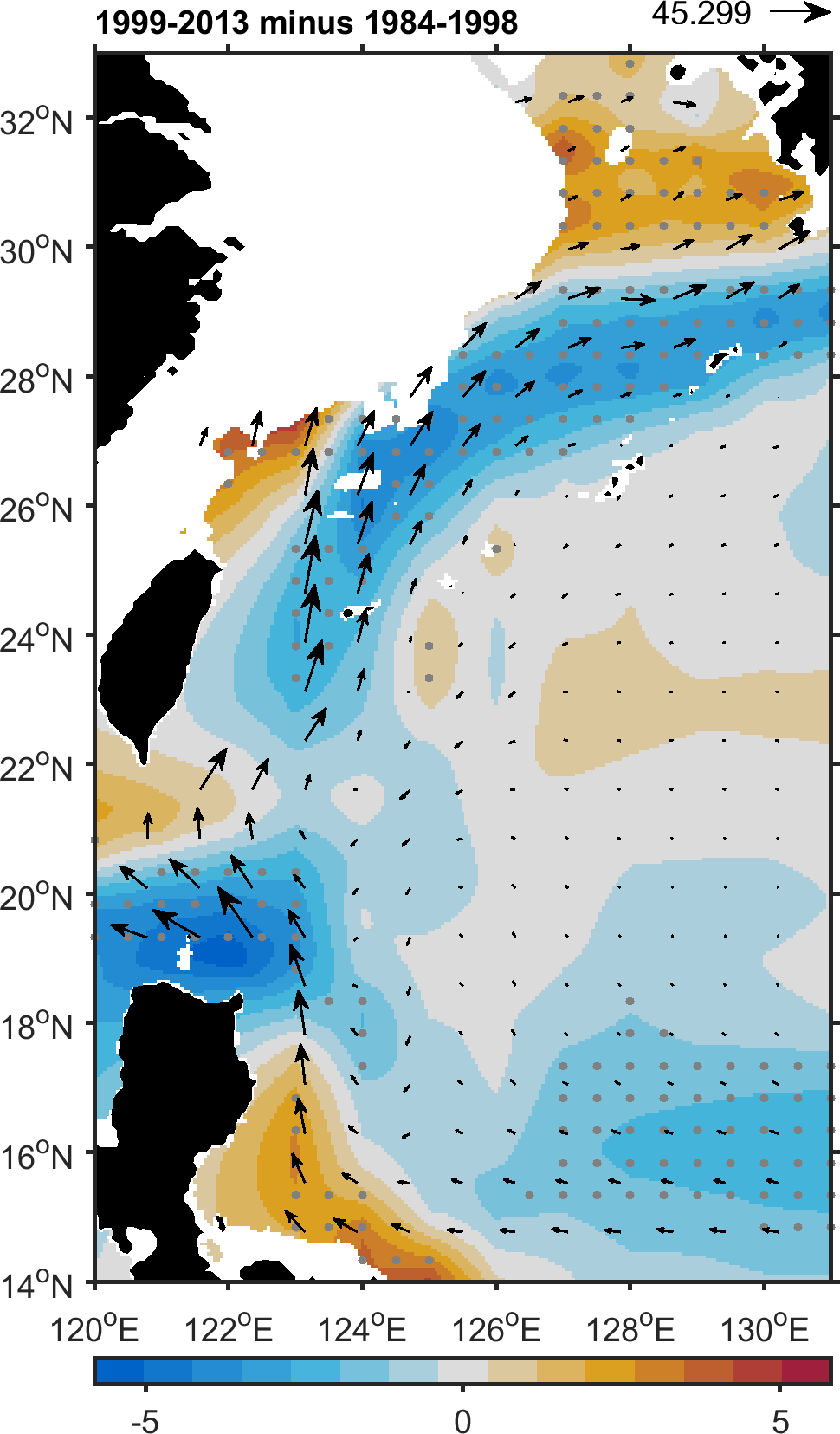


**Figure S3. Ocean surface speed difference (1999-2013 minus 1984-1998) based on GODAS.**

Gray dots indicate statistical significance above the 99% confidence level.


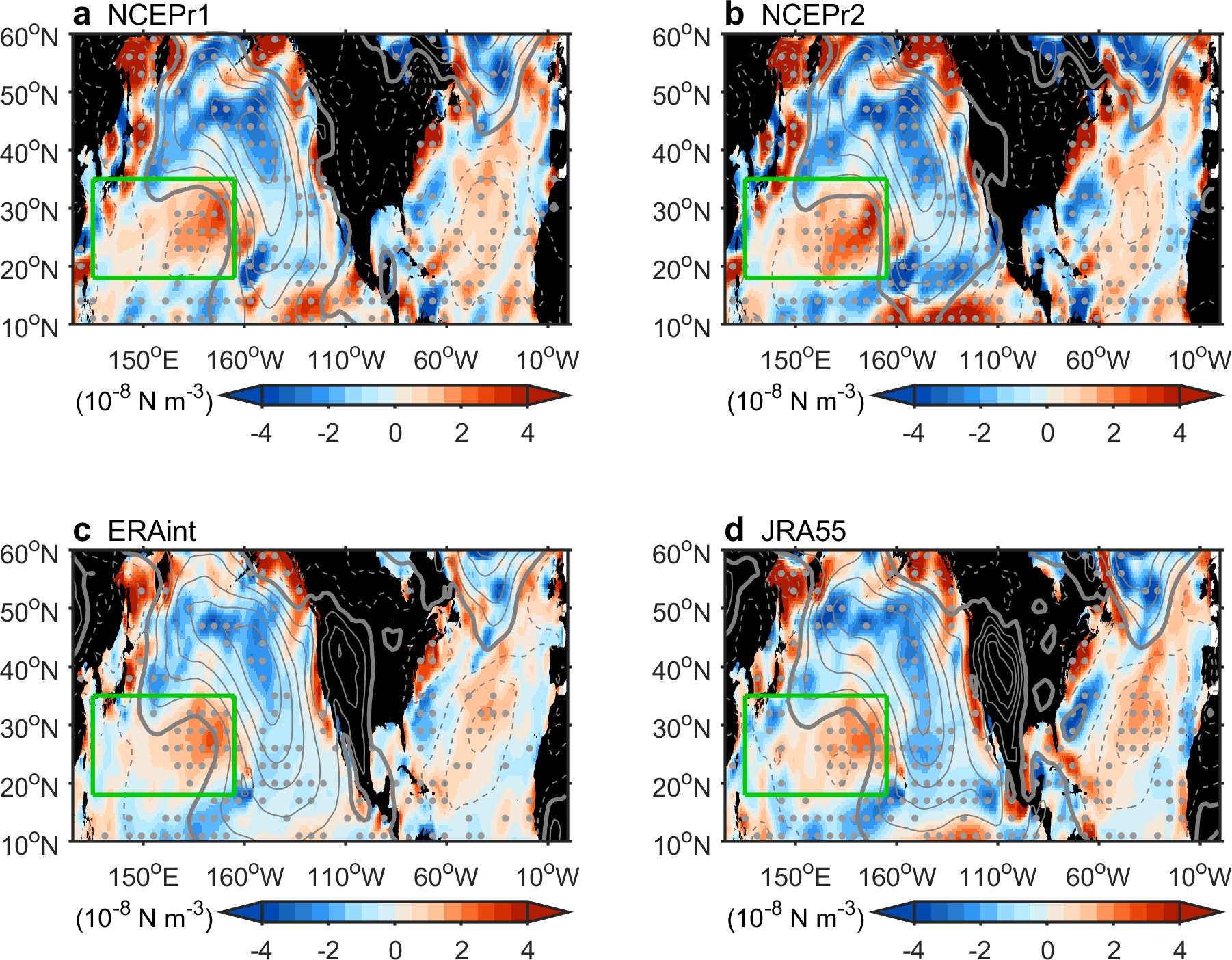


**Figure S4. Wind stress curl difference (shading) and sea level pressure difference (contour) (1999-2013 minus 1984-1998) based on various data sets.**

The green box indicates the location of the subtropical gyre. Gray dots indicate statistical significance above the 99% confidence level.


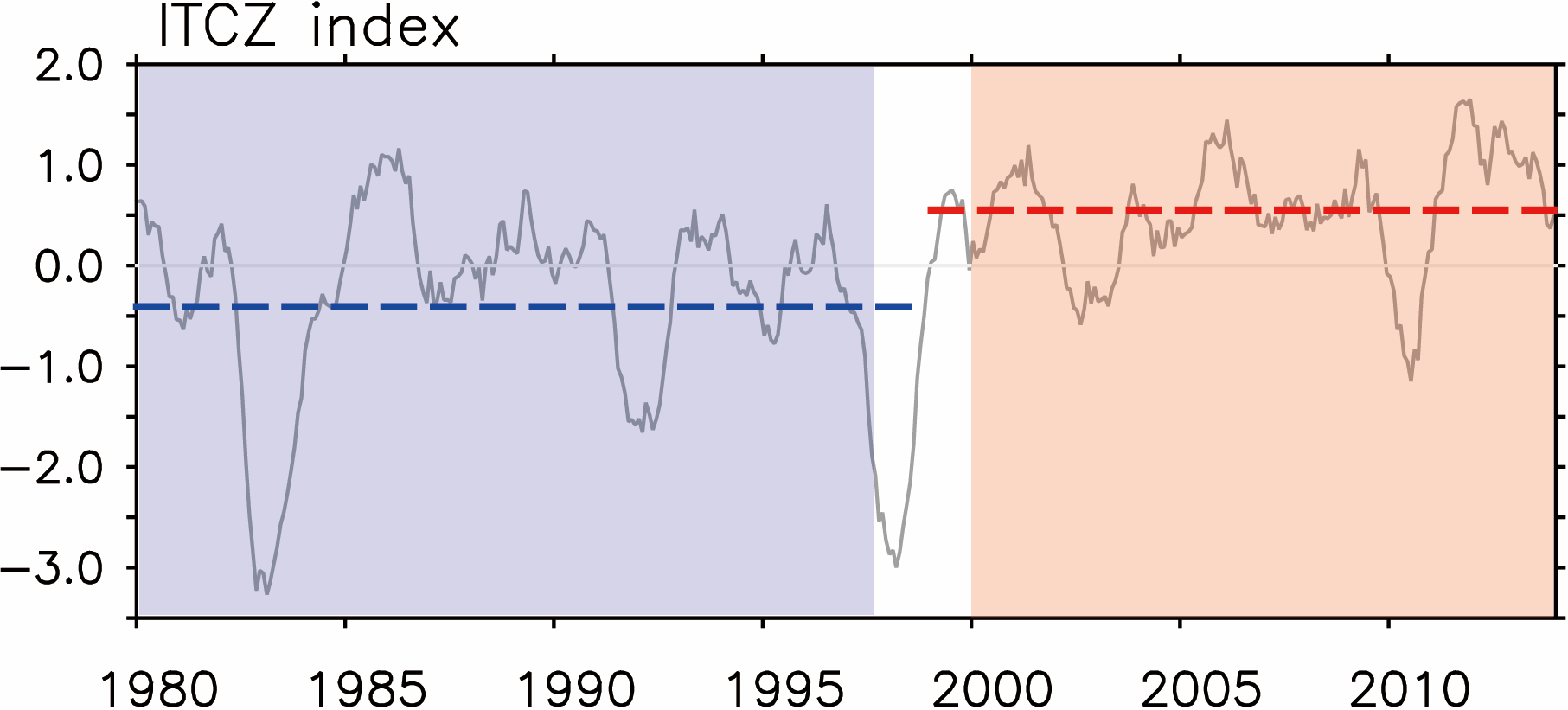


**Figure S5. Monthly time series of the ITCZ index (in units of mm day^-1^)**.

The AMO positive (negative) periods are denoted by the red (blue) shading. Red (blue) dashed lines indicate the means during the AMO positive (negative) periods.


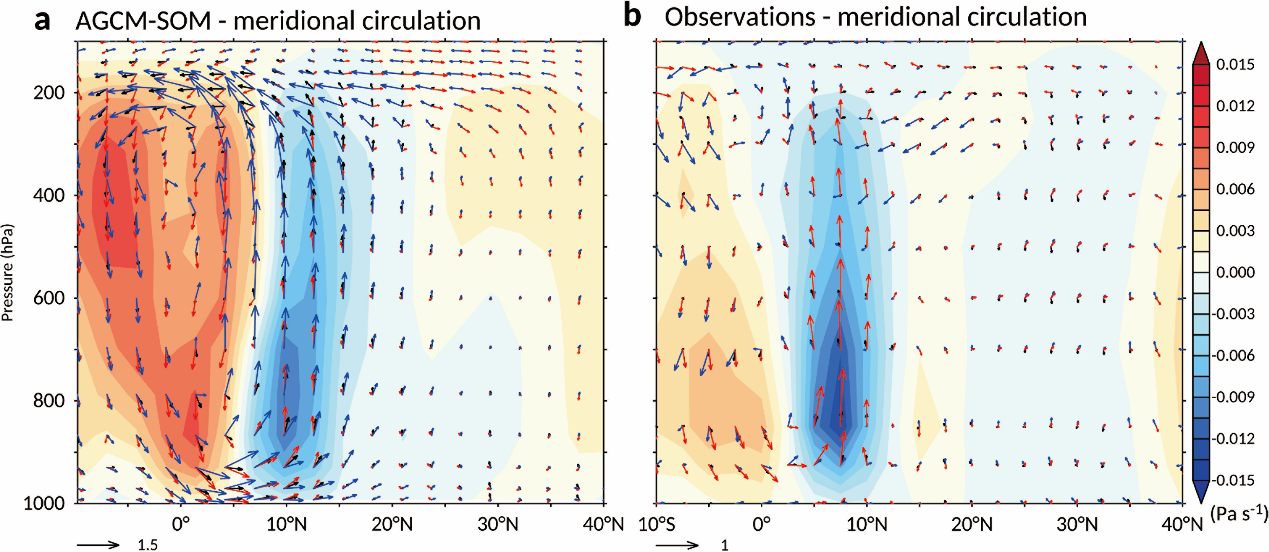


**Figure S6. Differences in the sector-averaged atmospheric meridional circulation between the AMO positive and negative phases base on (a) AGCM-SOM, and (b) observations (NCEPr1, NCEPr2, ERAint, and JRA55; 1999-2013 minus 1993-1998).**

Black, red, and blue vectors indicate the global, Pacific (120°E-90°W), and Atlantic (60°W-0) averages. The vertical velocities are multiplied by -100. The shading indicates the vertical velocities (unit in Pa s^-1^) in the Pacific.

**Table S1. Differences of ocean circulation characteristics before and after the 1998-99 abrupt change.**

This table is related to Fig. 1, and all the differences are statistical significance above the 99% confidence level based on t-test, indicating drastic changes in oceanic and atmospheric environments take place around 1998-99.

|  | First Period | Second Period | Difference |
| --- | --- | --- | --- |
| Kuroshio-TK | 1984 ~ 1998 | 1999 ~ 2013 | -1.2 cm |
| KIS |  |  | +5.01 cm |
| NP-SSTA |  |  | +0.22 ^o^C |
| NEC | 1989 ~ 1998 | 1999 ~ 2008 | -9.23 cm |
| NP-SSHA |  |  | +4.05 cm |

**Data set.**

**Observations and reanalysis data.** The monthly-mean SSTs of the ERSST (Extended Reconstructed Sea Surface Temperature, version 5) were provided by the NCEI/NOAA (National Centers for Environmental Information/National Oceanic and Atmospheric Administration, https://data.nodc.noaa.gov) with 2° x 2° horizontal resolution since 1854^2^. The tide gauge data were provided by the UHSLC (University of Hawaii Sea Level Center, https://uhslc.soest.hawaii.edu/)^3^. The SSHAs of the Reconstructed Sea Level dataset (version 1) were provided from CCAR (Colorado Center for Astrodynamics Research, the University of Colorado) and distributed by the JPL/NASA (Jet Propulsion Laboratory/National Aeronautics and Space Administration, https://podaac.jpl.nasa.gov/) since 1958, with 0.5 degree in spatial resolution and 7-day in temporal resolution^4^. The daily absolute geostrophic velocity (GSV) products (version: DT-MADT two-sat) were produced by Ssalto/Duacs and distributed by the AVISO (Archiving, Validation and Interpretation of Satellite Oceanographic Data, http://www.aviso.altimetry.fr) on a global 0.25° grid since 1993.

Three ocean reanalysis products were used in this study, the HYCOM (Hybrid Coordinate Ocean Model)^5^, the JCOPE2 (Japan Coastal Ocean Predictability Experiment 2)^6^, and the GODAS^7^. The HYCOM consortium was supported by the Navy DSRC (DoD Supercomputing Resource Center), and the NOPP/GODAE (National Ocean Partnership Program/Global Ocean Data Assimilation Experiment), with 1/12 degree horizontal resolution (GLBu0.08 dataset: “reanalysis” version from 1992-2012). The daily JCOPE2 product was provided by the JAMSTEC (Japan Agency for Marine-Earth Science and Technology) beginning in 1993, and has a 1/12 degree horizontal resolution. The monthly GODAS product was provided by NCEP/NOAA beginning in 1979, and has a 1/3 to 1 degree horizontal resolution.

Four atmospheric reanalysis products were used, the NCEPr1 (NCEP/National Center for Atmospheric Research reanalysis 1)^8^, the NCEPr2 (reanalysis 2)^9^, the ERAint (European Center for Medium-Range Weather Forecasts Reanalysis Interim)^10^, and the JRA55 (Japanese 55-year Reanalysis)^11^. The monthly NCEPr1 and NCEPr2 products are provided on a global 1.875° grid for the years 1948 (1979) to the present for the NCEPr1 (NCEPr2). The monthly ERAint product with an 80 km horizontal resolution is available from 1979 to the present. The monthly JRA55 was provided by the JMA (Japan Meteorological Agency), has a 55 km horizontal resolution and covers the period from 1958 to the present.

The Monthly precipitation values are obtained from the Global Precipitation Climatology Project Version 2.2 (GPCP V2.2, http://www.esrl.noaa.gov/psd/data/gridded/data.gpcp.html)^12^, which is based on a blend of satellite and in-situ measurements since 1979 on a global grid with a horizontal resolution of 2.5° × 2.5°.

**Model simulations.** Numerical model experiments were performed using the NCAR Community Atmospheric Model, version 3.0, (CAM3.0)^13^ with a T42 Eulerian spectral resolution (128 × 64 grid points) and 26 vertical levels. Model experiments were carried out with the prescribed AMO-associated SSTs in the North Atlantic (NA) (0°–70°N). In this set of the experiments, the atmospheric general circulation model (AGCM) is coupled to a mixed layer slab ocean model (SOM) in other ocean basins, and referred to as the AGCM–SOM experiments. The AGCM–SOM was integrated for 120 years for each of the two experiments, and model output from the last 100 years were used for the analysis. Using only the last 40 years of the AGCM– SOM output gives very similar results. The simulated AMO responses were defined as the mean state differences between the AMO-positive and AMO-negative experiments.

**Climate indices.** The AMO index^14^ is obtained from the Physical Sciences Division (http://www.esrl.noaa.gov/psd/data/timeseries/AMO/) which is calculated as the detrended SSTAs averaged over the North Atlantic from the equator to the 70°N.

**Statistical analyses.** All the differences in Table S1 as well as Figs. 3 and 4 are statistical significance above the 99% confidence level based on *t-test*.

**References**

1. Lin, Y.-F., Wu, C.-R. & Han, Y.-S. A combination mode of climate variability responsible for extremely poor recruitment of the Japanese eel (*Anguilla japonica*). *Scientific Reports* **7**, 44469 (2017).
2. Huang, B. *et al.* Extended reconstructed sea surface temperature, version 5 (ERSSTv5): upgrades, validations, and intercomparisons. *J. Clim.* **30**, 8179-8205 (2017).
3. Caldwell, P. C., Merrifield, M. A., & Thompson, P. R. Sea level measured by tide gauges from global oceans — the Joint Archive for Sea Level holdings (NCEI Accession 0019568), *NOAA National Centers for Environmental Information*, Dataset, Version 5.5 (2015).
4. Hamlington, B. D., Leben, R. R., Strassburg, M. W. & Kim, K. Y. Cyclostationary empirical orthogonal function sea-level reconstruction. *Geosci. Data J.* **1**, 13-19 (2014).
5. Bleck, R., & Boudra, D. B. Initial testing of a numerical ocean circulation model using a hybrid (quasi-isopycnic) vertical coordinate. *J. Phys. Oceanogr.* **11**, 755-770 (1981).
6. Miyazawa, Y. *et al.* Water mass variability in the western North Pacific detected in a 15-year eddy resolving ocean reanalysis. *J. Oceanogr.* **65**, 737-756 (2009).
7. Behringer, D. W., Ji M. & Leetmaa, A. An improved coupled model for ENSO prediction and implications for ocean initialization. Part I: The ocean data assimilation system, *Mon. Weather Rev.* **126**, 1013-1021 (1998).
8. Kalnay, E. *et al.* The NCEP/NCAR 40-year reanalysis project. *Bull. Am. Meteorol. Soc.* **77**, 437-471 (1996)
9. Kanamitsu, M., W. *et al.* Potter NCEP–DOE AMIP-II reanalysis (R-2), *Bull. Am. Meteorol. Soc.* **83**, 1631-1643 (2002).
10. Dee, D. P., *et al.* The ERA-Interim reanalysis: configuration and performance of the data assimilation system, *Q. J. R. Meteorol. Soc.* **137**, 553-597 (2011).
11. Kobayashi, S. *et al.* The JRA-55 reanalysis: general specifications and basic characteristics. *J. Meteor. Soc. Japan* **93**, 5-48 (2015).
12. Huffman, G. J. & Bolvin, D. T. GPCP Version 2.2 Combined Precipitation Data Set Documentation, Laboratory for Atmospheres, *NASA* **46** (2012).
13. Collins, W. D. *et al.* The formulation and atmospheric simulation of the Community Atmosphere Model version 3 (CAM3). *J. Clim.* **19**, 2144-2161 (2006).
14. Enfield, D. B., Mestas-Nunez, A. M. & Trimble, P. J. The Atlantic Multidecadal Oscillation and its relationship to rainfall and river flows in the continental US. *Geophys. Res. Lett.* **28**, 2077-2080 (2001).
